# Supplementary material for: Improvement of the Electronic—Neuronal Interface by Natural Deposition of ECM
Source: Materials (Basel). 2021 Mar 12;14(6):1378. doi: 10.3390/ma14061378 (PMC7999149; doi:10.3390/ma14061378)
Supplement: Supplementary file 1 [file materials-14-01378-s001.zip › Supplementary files/materials-1098683 SM final.docx]

Article

Improvement of the Electronic—Neuronal Interface by Natural Deposition of ECM

Tobias Weigel ^1,2,^*, Julian Brennecke ^2^ and Jan Hansmann ^1,2^

^1^ Translational Center for Regenerative Therapies, Fraunhofer Institute for Silicate Research ISC, 97082, Wuerzburg, Germany; info@isc.fraunhofer.de

^2^ Department Tissue Engineering and Regenerative Medicine, University Hospital Wuerzburg, 97070, Wuerzburg, Germany; lterm-assistenz@uni-wuerzburg.de

***** Correspondence: tobias.weigel@isc.fraunhofer.de


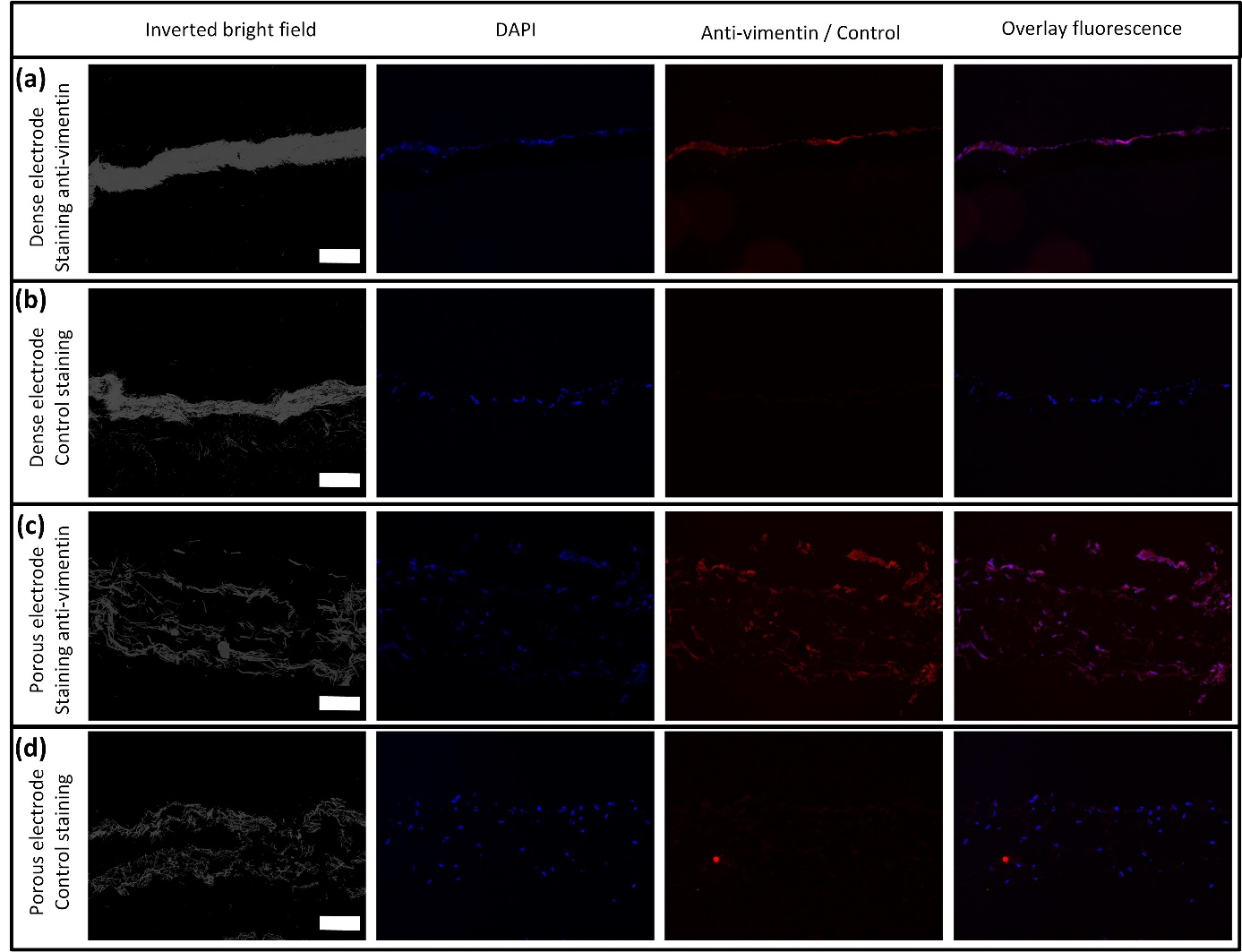


**Figure S1.** Immunofluorescence (IF) staining including control staining against vimentin of human dermal fibroblasts (hdf) after four weeks of culture. (**a**) IF staining against vimentin of hdf on the dense nano fiber electrode. (**b**) Control staining of the dense electrode with hdf. (**c**) IF staining against vimentin of hdf on the porous nano fiber electrode. (**d**) Control staining of the porous electrode with hdf. Scale bars correlate to 100 µm.


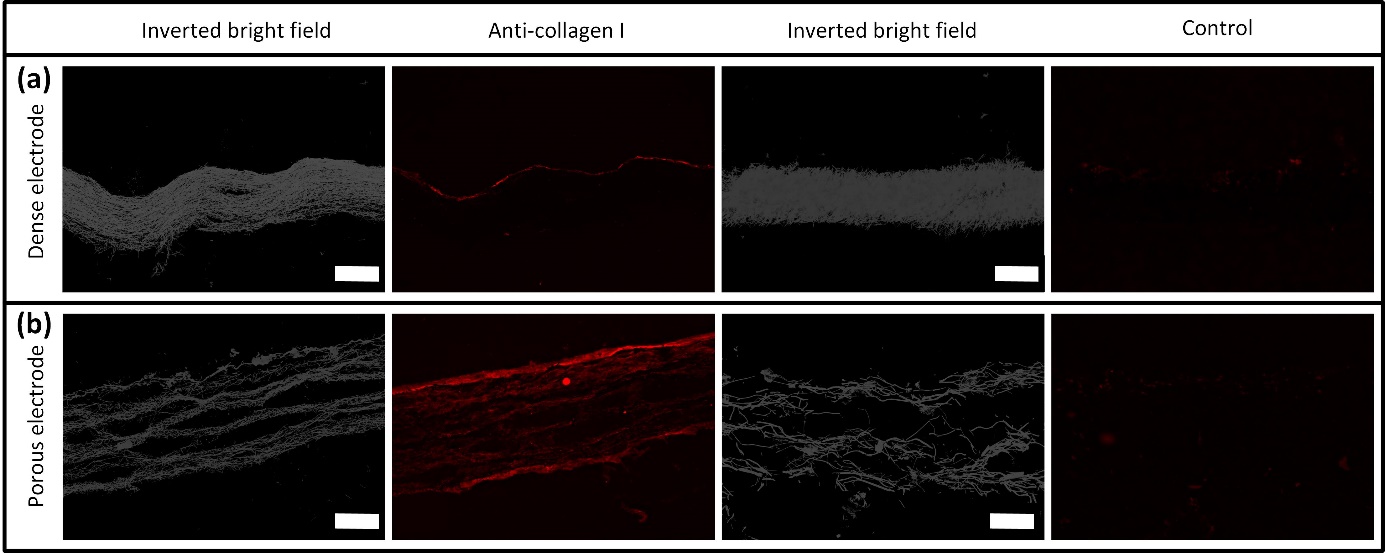


**Figure S2.** Immunofluorescence (IF) staining including control staining against collagen I after removing the cultured hdf (4 weeks) with sodium desoxycholate. (**a**; left pictures) IF staining against collagen I of the generated ECM on the dense nano fiber electrode . (a; right picture) Control staining of the dense electrode with ECM. (**b**; left pictures) IF staining against collagen I of the generated ECM on the porous nano fiber electrode . (b; right picture) Control staining of the porous electrode with ECM. Scale bars correlate to 100 µm.
